# Supplementary material for: DNA methylation signatures of frailty beyond age: a longitudinal study of female and male mice
Source: bioRxiv. 2025 Dec 16:2025.12.13.694109. Preprint. [Version 1] doi: 10.64898/2025.12.13.694109 (PMC12724424; doi:10.64898/2025.12.13.694109)
Supplement: Supplement 2 [file NIHPP2025.12.13.694109v1-supplement-2.pdf]

bioRxiv preprint doi: https://doi.org/10.64898/2025.12.13.694109; this version posted December 16, 2025. The copyright holder for this preprint (which was not certified by peer review) is the author/funder, who has granted bioRxiv a license to display the preprint in perpetuity. It is made available under aCC-BY 4.0 International license.

Supplementary Information

Supplementary Fig.1. Distribution of frailty index. Frailty index (FI) for each mouse at each time point was derived from the 31-Item Clinical Frailty Index test. **a.** Boxplot of FI in the discovery cohort stratified by sex and time points (age in months). **b.** Boxplot of FI in the validation cohort stratified by sex and time points (age in months). The box represents the interquartile range from the 25th to the 75th percentile, the line inside the box indicates the median (50th percentile). Outliers (beyond 1.5 times the interquartile range) are plotted as individual points.

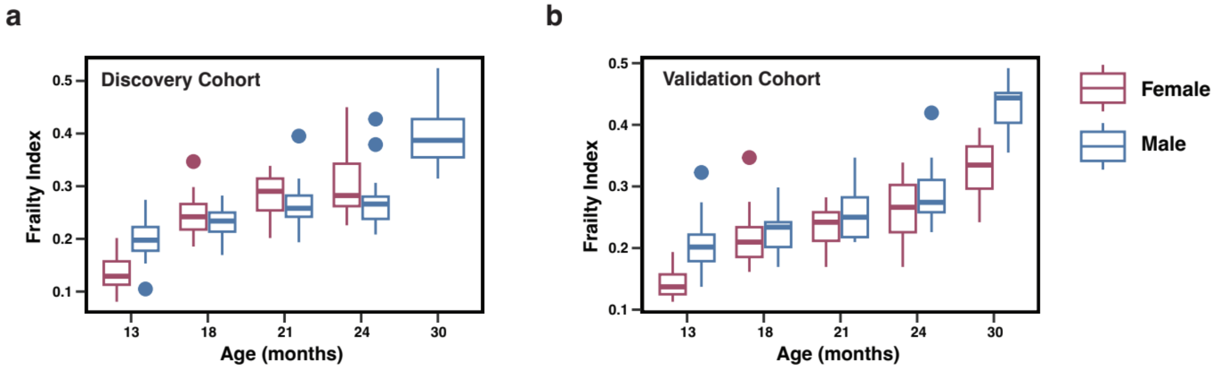

bioRxiv preprint doi: https://doi.org/10.64898/2025.12.13.694109; this version posted December 16, 2025. The copyright holder for this preprint (which was not certified by peer review) is the author/funder, who has granted bioRxiv a license to display the preprint in perpetuity. It is made available under aCC-BY 4.0 International license.

Supplementary Fig.2. Global DNA methylation changes across time and sex. DNA from peripheral blood mononuclear cell samples in the discovery cohort (*n* = 153) from 20 female and 25 male mice was analyzed using a DNA methylation array. After quality control and filtering, 242,599 CpG sites were retained for downstream analysis. **a.** Distribution of beta values by density plot stratified by sex and age (13 mo, 18 mo, 21 mo, 24 mo, and 30 mo). **b.** Distribution of beta values by violin and boxplot stratified by sex and age. Dashed lines connect the median beta values across time points to illustrate temporal trends in global methylation levels. **c.** Principal Component analysis (PCA) plot (PC1 vs. PC2) showing sample variation by sex and time point. Analysis was performed on M-values (logit-transformed beta values). **d.** PCA plot (PC1 vs. PC2) showing potential associations between methylation patterns and frailty (log-transformed frailty index, $\log_2$(FI)). **e.** Heatmap showing total sample variance and PC variance (%) explained by mouse ID, sex, age and FI residual (age effects regressed out). Variance partitioning analysis was performed using linear mixed models to estimate the variance attributable to variables.

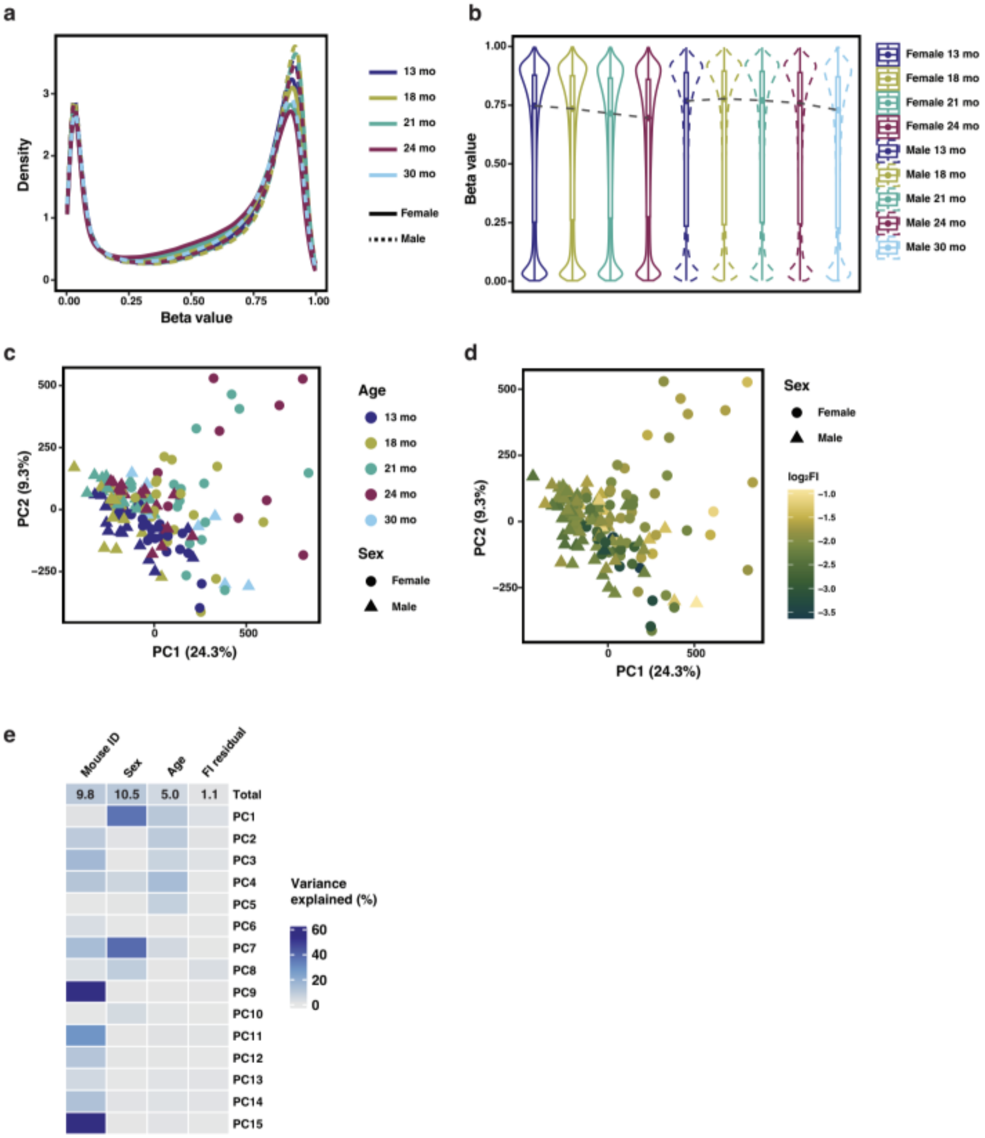

bioRxiv preprint doi: https://doi.org/10.64898/2025.12.13.694109; this version posted December 16, 2025. The copyright holder for this preprint (which was not certified by peer review) is the author/funder, who has granted bioRxiv a license to display the preprint in perpetuity. It is made available under aCC-BY 4.0 International license.

Supplementary Fig.3. Visualization and characterization of frailty-related DMPs and DMRs in sex-inclusive subgroup. a. Manhattan plot showing the differentially methylated positions (DMPs) associated with frailty. DMPs were selected by controlling for a 5% Benjamini-Hochberg (BH) false discovery rate. **b.** Barplot showing the proportion of CpG and gene location categories. Proportions of 'Island' and 'TSS200' among all CpGs included in the analysis were compared to those of DMPs in all chromosomes, autosomes and X chromosomes by chi-square test, with *** for $p < 0.001$. **c.** Manhattan plot showing the differentially methylated regions (DMRs) associated with frailty. DMRs were identified by Stouffer's Z-score method and selected based on a p-value threshold of 0.05. The size of each point corresponds to the number of CpGs included. **d.** Scatter plot showing p-values of DMR in associations with age-independent (associated with frailty adjusting for age) and age-dependent frailty. Each point represents a DMR, and is colored according to classification. **e.** Heatmap showing association between modules with frailty and age. Module eigenvalues were subjected to association with log-transformed FI (adjusted for sex or adjusted for sex and age) and age by applying linear mixed models. Significance was determined by p-values at a cutoff of 0.05, with * for $p < 0.05$, ** for $p < 0.01$, and, *** for $p < 0.001$. **f** and **g.** Illustrative examples of differentially methylated regions. Each point represents the methylation beta value of a CpG within the genomic location and CpGs from the same sample are connected by smoothed lines fitted using the LOESS method. Both points and lines are colored according to the level of log-transformed frailty index ($\log_2$FI). Distribution of CpG location categories (colored according to the category) and exons is presented in panels.

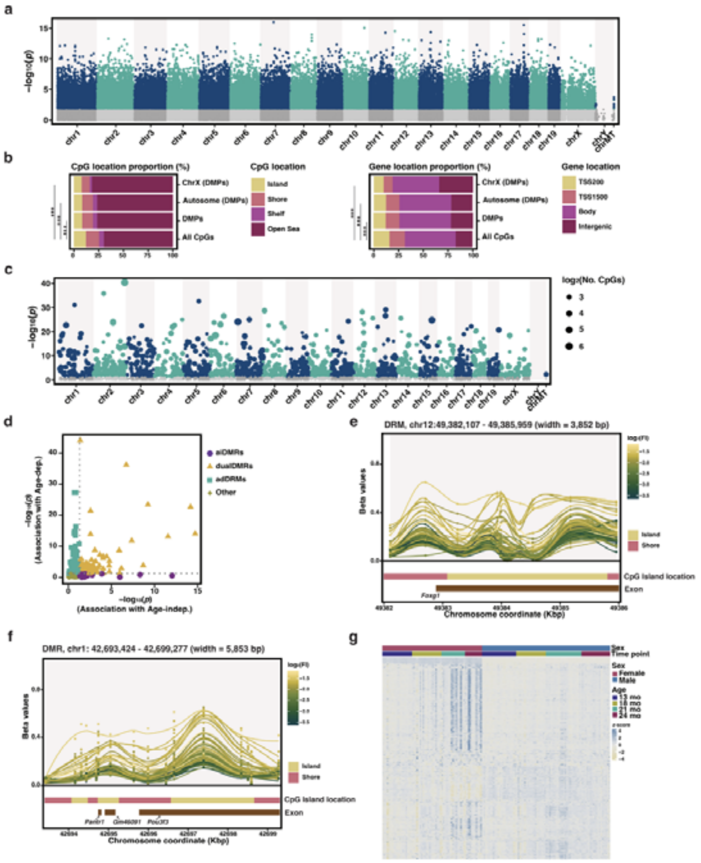

bioRxiv preprint doi: https://doi.org/10.64898/2025.12.13.694109; this version posted December 16, 2025. The copyright holder for this preprint (which was not certified by peer review) is the author/funder, who has granted bioRxiv a license to display the preprint in perpetuity. It is made available under aCC-BY 4.0 International license.

Supplementary Fig.4. Selection of CpGs linked to X chromosome inactivation and escape. a. Manhattan plot showing the differentially methylated positions (DMPs) associated with frailty in females and males. DMPs were selected by controlling for a 5% Benjamini-Hochberg (BH) false discovery rate. **b.** Barplot showing the proportion of CpG and gene location categories in females (F.) and males (M.). Comparisons of proportions of 'Island' and 'TSS200' among all CpGs included in the analysis (All CpGs), DMPs in all chromosomes, autosomes (at.) and X chromosomes (chrX) were performed by chi-square test, with ** for $p < 0.01$ and *** for $p < 0.001$. **c.** Distribution of beta values of X chromosome linked CpGs in females and males. **d.** Identification of CpGs types related to X chromosome inactivation. CpGs were classified by beta value difference and overlap of CpG methylation ranges between females and males. CpG types are colored and shaped according to the classification (escape, X chromosome inactivation escape).

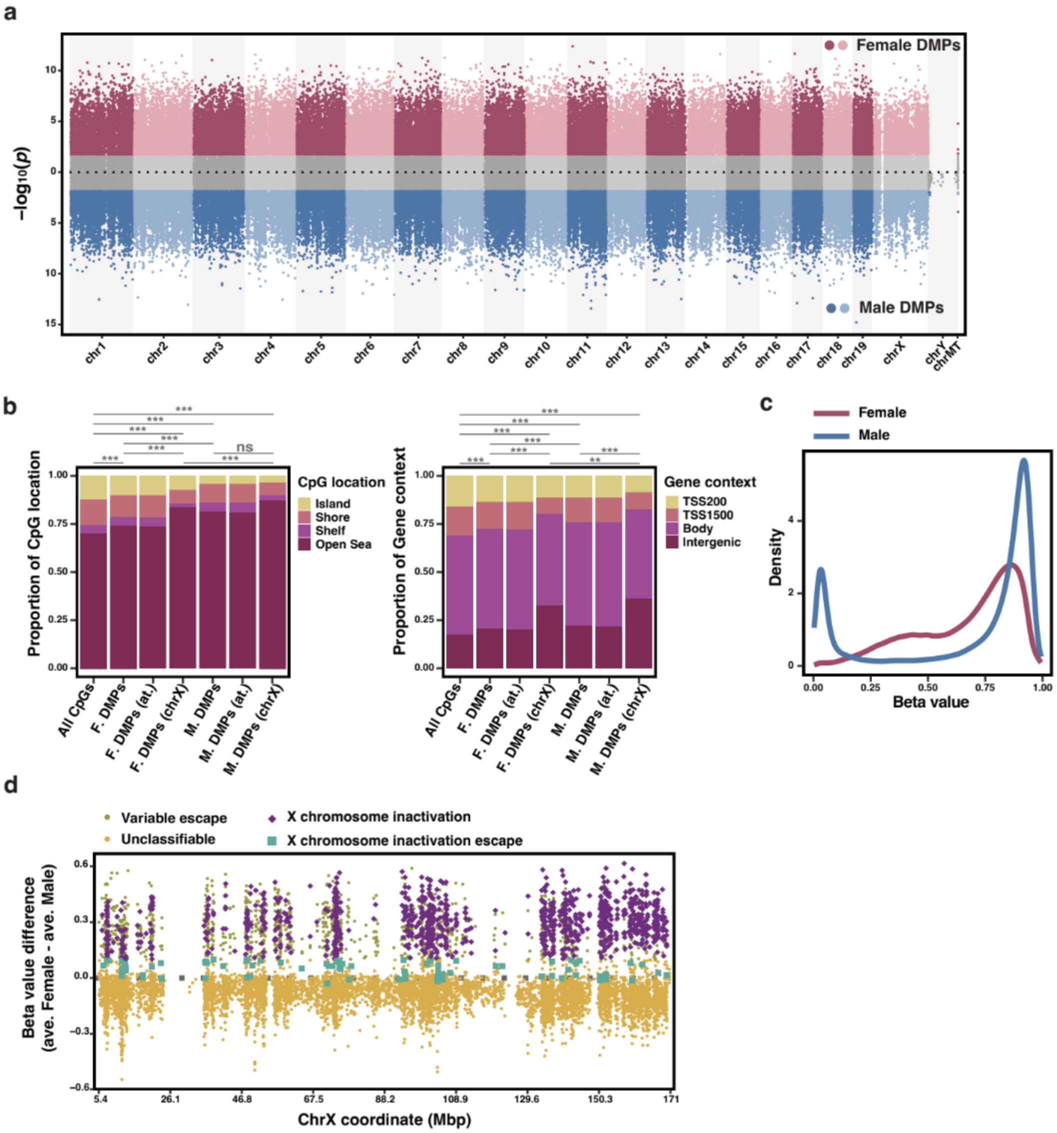

bioRxiv preprint doi: https://doi.org/10.64898/2025.12.13.694109; this version posted December 16, 2025. The copyright holder for this preprint (which was not certified by peer review) is the author/funder, who has granted bioRxiv a license to display the preprint in perpetuity. It is made available under aCC-BY 4.0 International license.

Supplementary Fig.5. Visualization and characterization of DMRs in females and males. **a.** Manhattan plot showing the differentially methylated regions (DMRs) associated with frailty in females. DMRs were identified by Stouffer's Z-score method and selected based on a *p*-value threshold of 0.05. The size of each point corresponds to the number of CpGs included. **b.** Scatter plot showing *p*-values of association between DMRs with age-indep. and age-dep. frailty in females. **c.** Illustrative examples of differentially methylated regions. Each point represents the methylation beta value of a CpG within the genomic location and CpGs from the same sample are connected by smoothed lines fitted using the LOESS method. Both points and lines are colored according to the level of log-transformed frailty index ($\log_2$FI). Distribution of CpG location categories (colored according to the category) and exons is presented in panels. **d.** Manhattan plot showing the differentially methylated regions (DMRs) associated with frailty in males. **e.** Scatter plot showing *p*-values of association between DMRs with age-indep. and age-dep. frailty in males. **f.** and **g.** Examples of DMRs that present sex-specific associations with frailty.

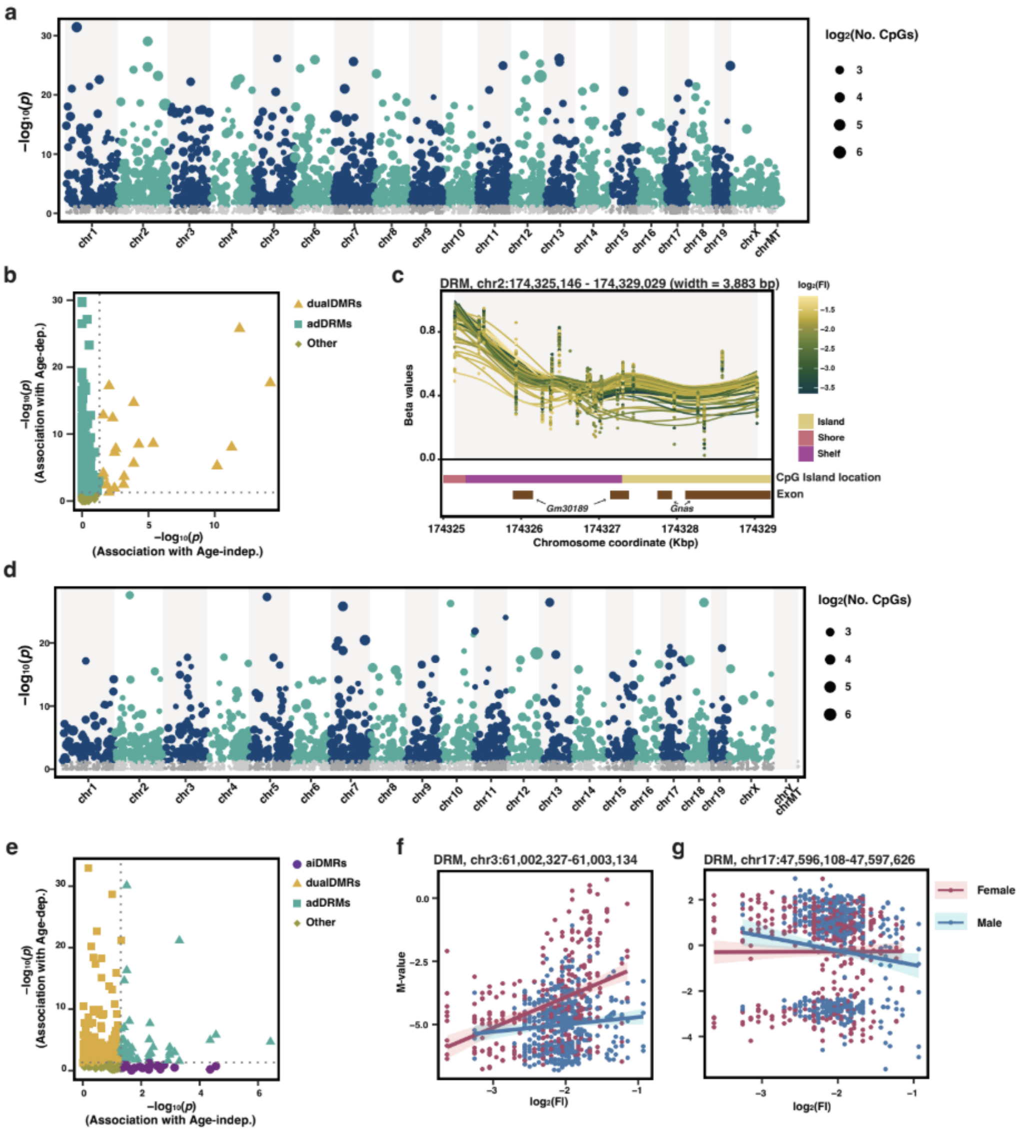

bioRxiv preprint doi: https://doi.org/10.64898/2025.12.13.694109; this version posted December 16, 2025. The copyright holder for this preprint (which was not certified by peer review) is the author/funder, who has granted bioRxiv a license to display the preprint in perpetuity. It is made available under aCC-BY 4.0 International license.

Supplementary Fig.6. Examples of DMRs that present positive and negative effect sizes in association with frailty.

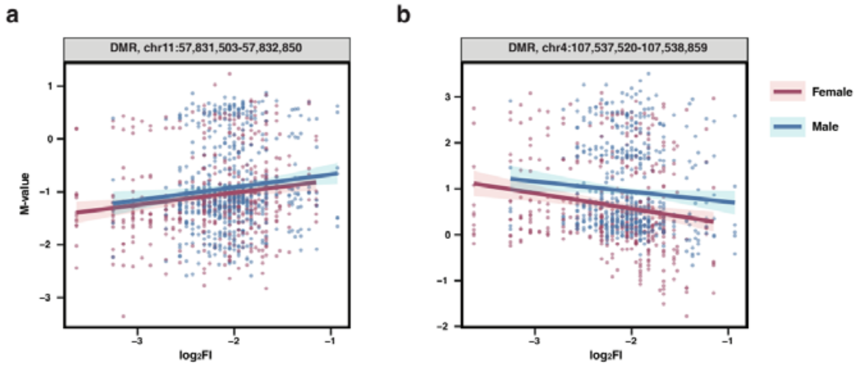

Supplementary Fig.7. Partial regression plot showing example frailty-related DMRs association with frailty outcomes. a. The DMR presents opposite directions when associated with two future frailty outcomes. M-values of or changes of M-value from a prior time point (□M) for each CpG within the DMR were used as the independent variable and outcomes included $logFI_c$ (log-transformed Frailty Index at Age_c), $logFI_f$ (log-transformed FI at a future age) and □logFI (the difference between $logFI_f$ and $logFI_c$). For $logFI_f$, when used as the outcome to be associated with ΔM, further adjustment for $logFI_c$ was performed. **b.** The DMR presents opposite directions when associated with □logFI (ΔM as the variate) across the discovery and validation cohorts.

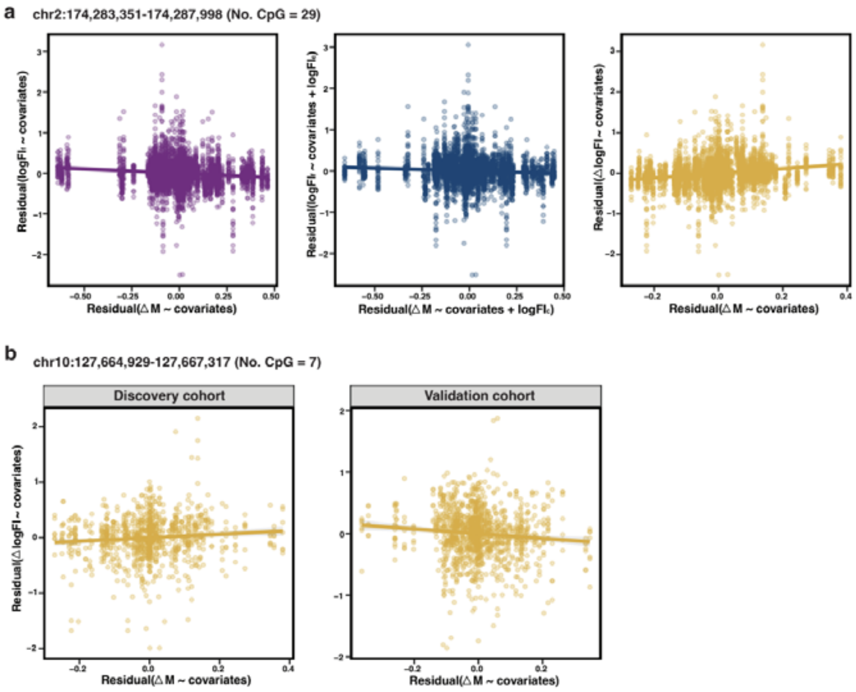

bioRxiv preprint doi: https://doi.org/10.64898/2025.12.13.694109; this version posted December 16, 2025. The copyright holder for this preprint (which was not certified by peer review) is the author/funder, who has granted bioRxiv a license to display the preprint in perpetuity. It is made available under aCC-BY 4.0 International license.

Supplementary Fig.8. Variability analysis of frailty-related CpGs. a. Violin plot showing the total intercept variance stratified by the DMR categories the CpGs belong to in sex-inclusive and sex-specific groups. Variance was estimated using linear mixed models with random intercepts and slopes grouped by individual mice. **b.** Violin plot showing the total variance of slopes stratified by the DMR categories the CpGs belong to in sex-inclusive and sex-specific groups. **c.** Examples of DMRs that present similar slopes but distinct intercept in individuals. **d.** An example of DMRs that present deterministic methylation changes in frailty progression. **e.** An example of DMRs that present stochastic methylation changes in frailty progression. **f-h.** Scatter plots showing Shannon entropy of CpGs belonging to different DMR categories in frailty progression in sex-inclusive and sex-specific groups. Shannon entropy values were calculated using adjusted beta values, with sex effects regressed out, for each CpG within a DMR.

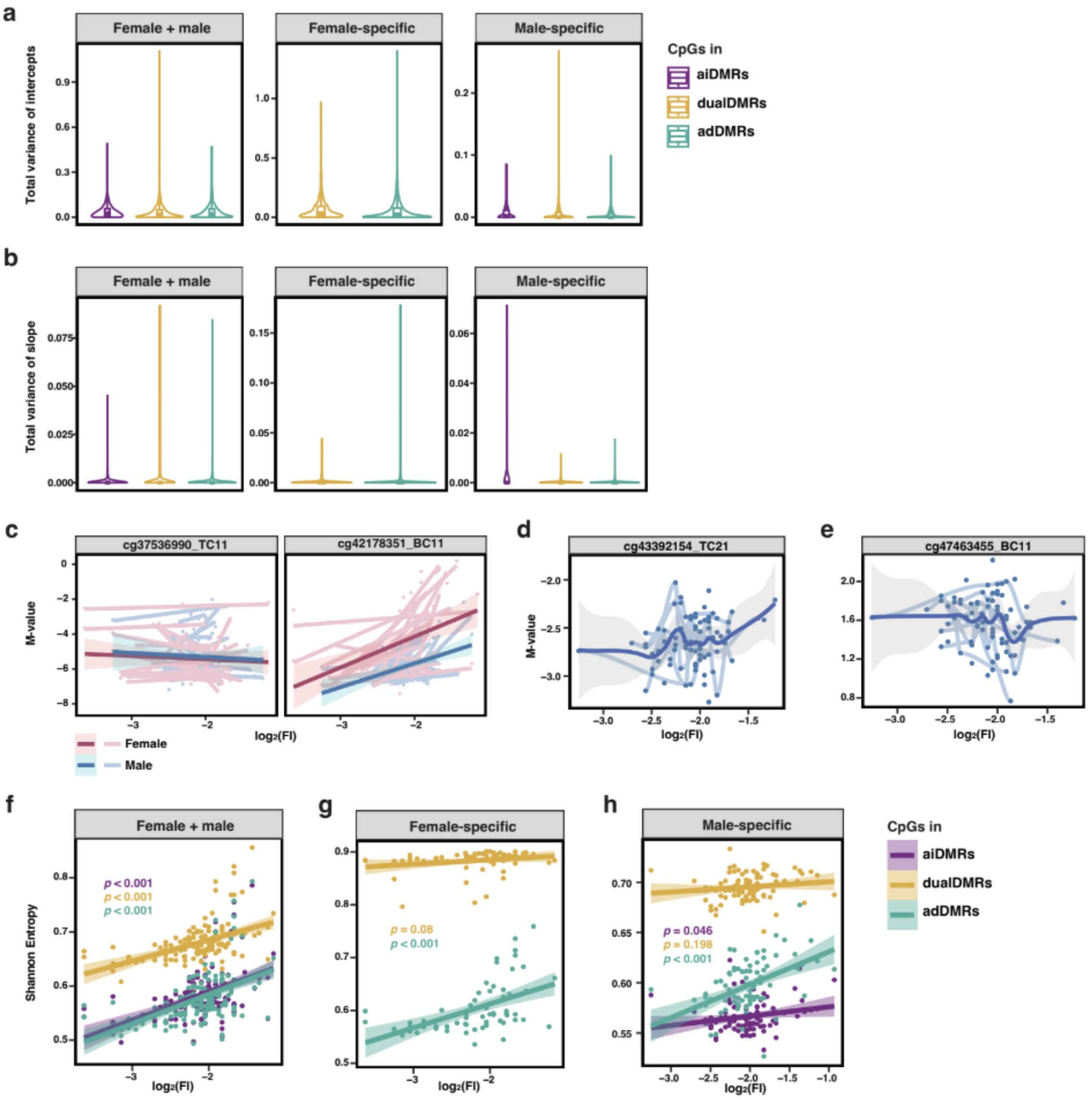

bioRxiv preprint doi: https://doi.org/10.64898/2025.12.13.694109; this version posted December 16, 2025. The copyright holder for this preprint (which was not certified by peer review) is the author/funder, who has granted bioRxiv a license to display the preprint in perpetuity. It is made available under aCC-BY 4.0 International license.

Supplementary Fig.9. Comparisons of models that include diverse combinations of variables. The optimal number of top ranking DMRs (ranked by absolute effect size in association with frailty) were selected based on RMSE.

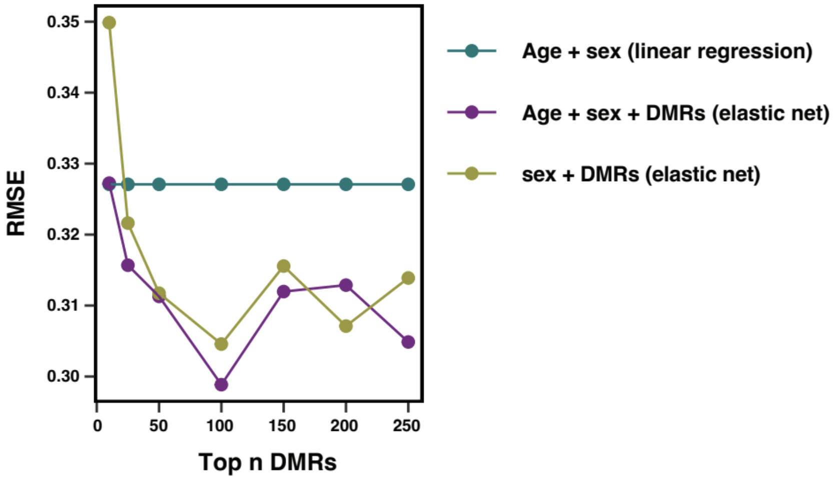

Supplementary Fig.10. Comparisons of the performance of the epigenetic frailty clock and age+sex linear regression model in mice at the same age.

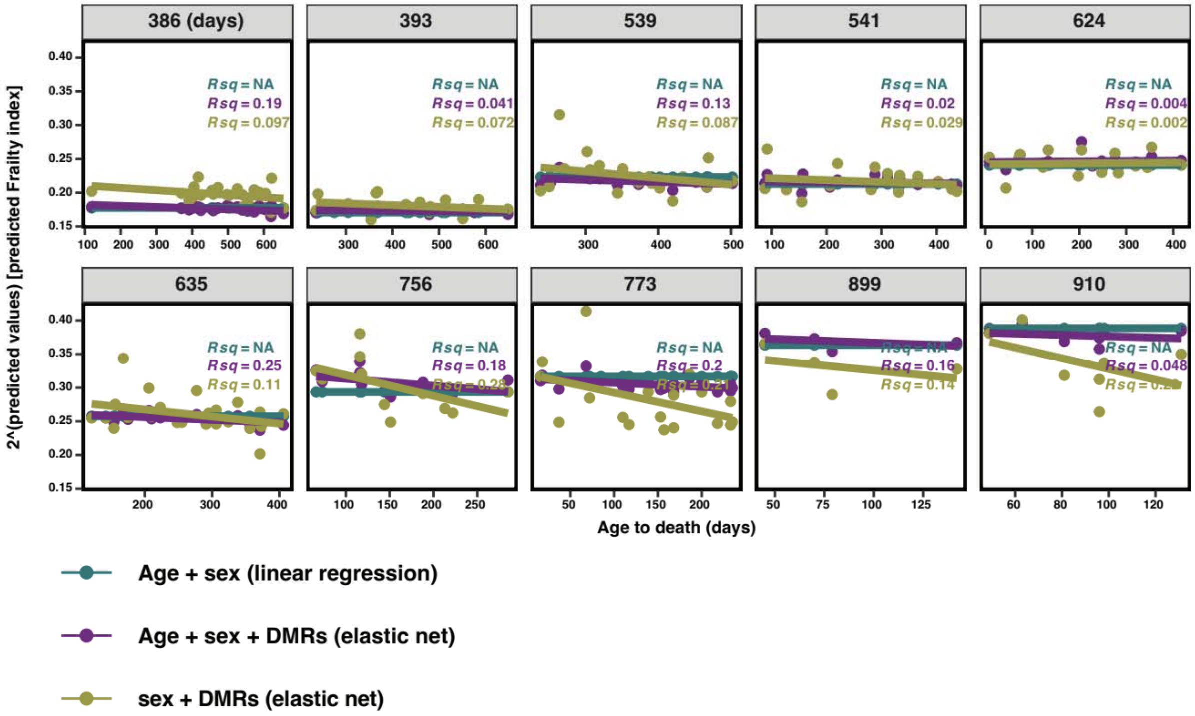

Supplementary Tables1-9

https://docs.google.com/spreadsheets/d/1gMrRaDpwGZ2D3VctHy9Cx-Pw1HuQMNE7_Sb1aOQD-Cg/edit?gid=109275884#gid=109275884
